# Supplementary material for: Detection and localization of conspecifics in ghost knifefish are influenced by the relationship between the spatial organization of receptors and signals
Source: Front Comput Neurosci. 2026 Jun 23;20:1837263. doi: 10.3389/fncom.2026.1837263 (PMC13337657; doi:10.3389/fncom.2026.1837263)
Supplement: Supplementary file 1 [file Data_Sheet_1.docx]

**Supplementary Information**

**Table S1: Model parameters used for the prototypic seed neuron (see Methods for details).**

| Description | Name | Value | Description | Name | Value |
| --- | --- | --- | --- | --- | --- |
| Membrane time constant (s) | *τ_m_* | 15·10^-4^ | Noise strength (A) | *A_σ_* | 15·10^-9^ |
| Lean reversal potential (V) | *E_m_* | -70·10^-3^ | Adaptation reversal potential (V) | *E_α_* | -80·10^-3^ |
| Membrane resistance (Ω) | *R_m_* | 15·10^5^ | Adaptation increment (V) | *Δ_α_* | 14.5·10^-8^ |
| Spiking threshold (V) | *V_T_* | -49·10^-3^ | Adaptation time constant (s) | *τ_α_* | 50·10^-3^ |
| Reset potential (V) | *V_R_* | -70·10^-3^ | EOD amplitude (A) | *A_EOD_* | 1.7·10^-7^ |
| Refractory period (s) | *t_R_* | 9·10^-4^ | EOD baseline bias (A) | *β* | 3·10^-8^ |


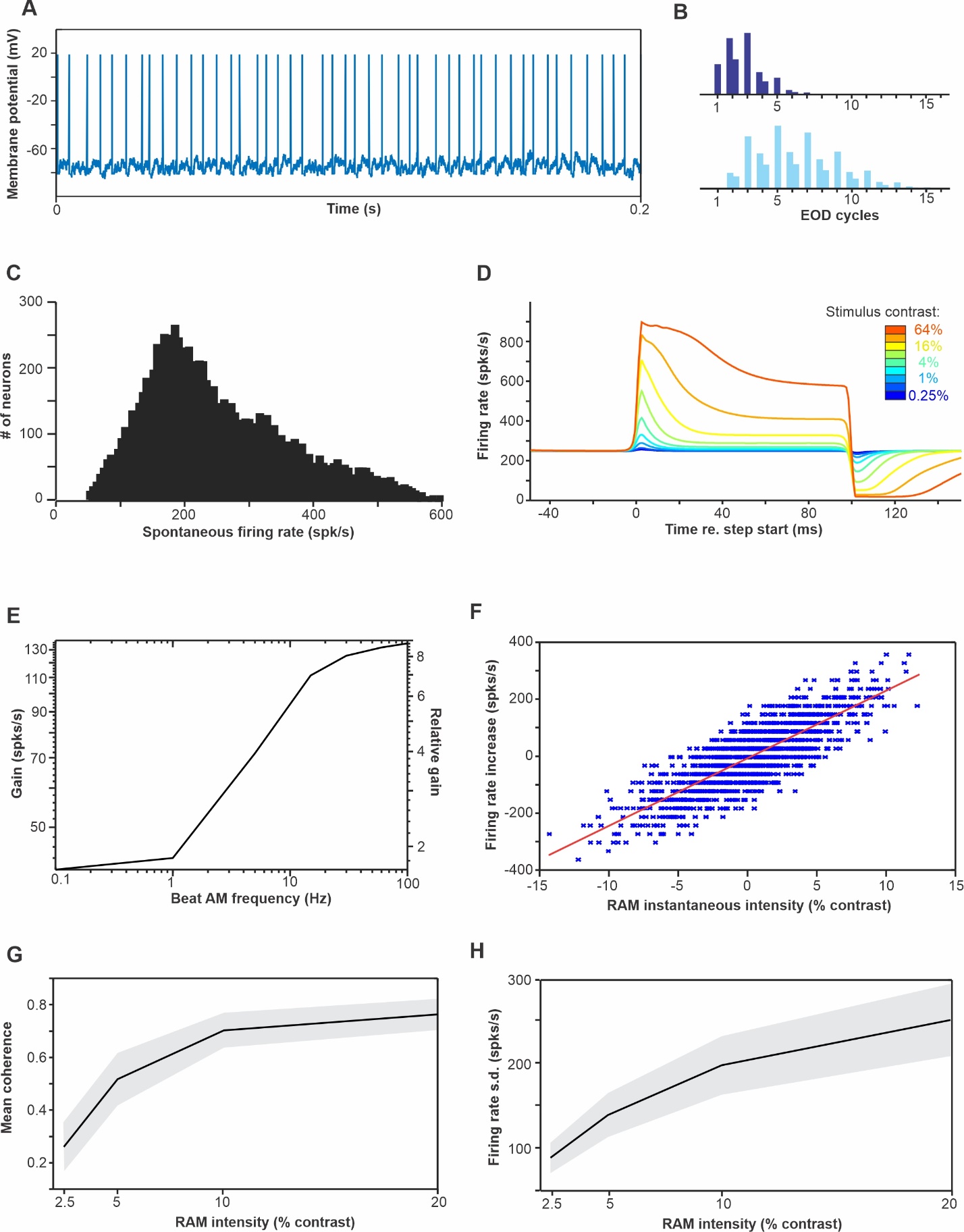


**Figure S1: Average response properties of the heterogeneous population of modeled receptors.**

**A.** an example of the membrane potential and spiking pattern of a model response (spontaneous activity). **B.** Inter-spike intervale histogram of 2 different model neurons (spontaneous activity) showing phase locking to the EOD period and different firing tendencies. Note that the x-axis is expressed in multiples of the EOD period but since we used an EOD frequency of 1,000 Hz for simplicity, this also corresponds to ms. **C.** Distribution of spontaneous firing rate across our entire population. This distribution was achieved by selecting 26 seed neurons with firing rates unevenly distributed along this range and diversifying model parameters based on these seeds (see Methods). This range and distribution replicates published data (Bastian, 1981; Ratnam and Nelson, 2000; Grewe et al., 2017). In particular, the mean spontaneous firing rate was 251 spk/s with a CV of 0.45. **D.** Responses to step increases in EOD intensity. The strength of the peak response, the steady-state response, and the adaptation time course were matched to published data (Benda et al., 2005). **E.** Response gain to beat stimuli of different AM frequencies. Although we did not explore systematically the response of our model at different beat frequencies in the results section, we calculated the gain for a range of AM frequencies. This analysis helps us to evaluate the sensitivity of the neurons (see the absolute scale on the left) and it also helps to assert that the adaptation dynamic replicates some of the tuning properties of the neurons (see the relative scale on the right; gain for an AM of 1 Hz is normalized to 1). This average gain curve is comparable to experimental data (Nelson et al., 1997; Chacron et al., 2005). **F.** Relative firing rate during random amplitude modulations. We replicated a published analysis of receptor sensitivity (Gussin et al., 2007a) that measures the relative firing rate (relative to average) in successive 32 ms windows during the response to a low frequency (0 to 4 Hz) random amplitude modulation. We note that our scale is different from theirs: our ± 5% values correspond to the absolute contrast of the stimulus (i.e. s.d. of 10% contrast) whereas the scale in their Fig 3 has ± 50% being the min-to-max of their 10% contrast stimulus. When converted to the same scale, we find a gain slope of 23.7 spk/s/% (± 5.8 s.d.) comparable to the 17.7 spk/s/% (range 3.2 to 40.2) they found. **G.** Mean coherence at 10-30 Hz for random amplitude modulations (0-100 Hz) of different overall intensities (contrast). We qualitatively matched the coherence to values found in previous publications(Chacron et al., 2005; Grewe et al., 2017). **H.** Firing rate modulation in response to random amplitude modulations (0-300 Hz). We replicated the analysis in Grewe et al. (2017; see their figure S1A) which plots the standard deviation of the response (averaged over trials) as a function of stimulus contrast. Their population averages go from approximately 100 spk/s at 2.5% contrast to 200 spk/s at 20% contrast with a large variability among the population; we have a population average between 90 spk/s and 250 spk/s respectively.


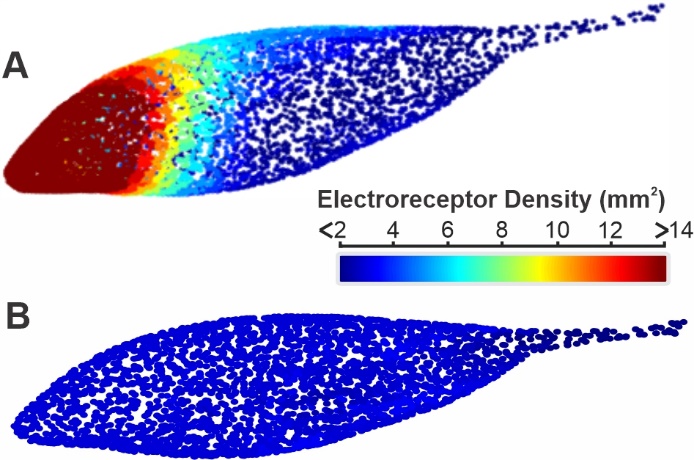


**Figure S2:** **Receptor density compared between our full population (A) and our uniformly low-density population (B)**. Each dot shows the position of a receptor and the color reflects the density of receptors at this location. The uniform population was created by selecting, for each face of the mesh model, a subset of receptors from the full population to match the density of 2 receptors per mm^2^.


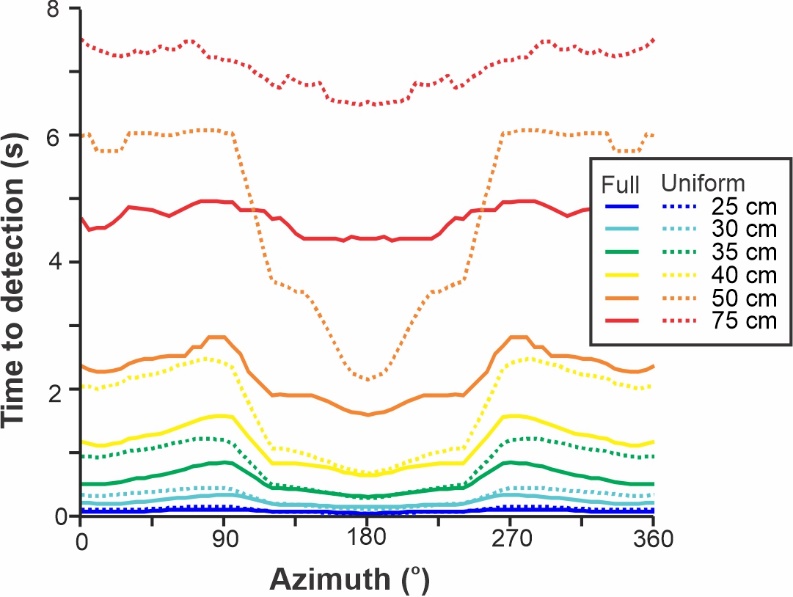


**Figure S3: Detection performance as a function of source location and receptor structure.** We compare a full population of receptors that includes a high density in rostral regions with a population that has a uniform density across the body matching the low density of the caudal region of the body (see Figure S2B). We present here the results for distances above 25 cm where we can see differences across azimuth and population structures. Our decoder considers that the response strength of receptors can be integrated across time and thus more noisy, weak responses require integration across longer periods to reach a reliable detection performance. We plot here the integration time required for our decoder to reach 95% detection accuracy and plot this here as a function of stimulus location.
